# London Letter

**Published:** 1887-08

**Authors:** Wm. B. Meany

**Affiliations:** London, England


### FOREIGN CORRESPONDENCE.

*To the Editor of the Chicago Medical Journal and Examiner:*

THE Fifty-fifth Annual Meeting of the British Medical Association was held in Dublin during the first week of August of this year. The membership of the Association now numbers 12,000, having increased four fold in twenty years. The president for this year is Dr. John T. Banks. His inaugural address was devoted chiefly to a discussion of matters of local interest. He declined to discuss the question of the expediency of women entering the medical profession. He said that the Royal University had no discretion in the matter, and that the College of Physicians and Surgeons had voluntarily thrown open its doors to, and and afforded them every opportunity. Last year the degree of B. A. was conferred upon nine women, four of them taking honors.

The work of the Association is done in sections.

In the section on

PUBLIC MEDICINE,

Sir Thomas Crawford, president of the section, said that diplomas in sanitary science, public health, etc., must sooner or later be recognized as one of the necessary qualifications of medical men. He thought it desirable that there should be uniformity of title as to the registration and qualification of medical men under this head. It was by no means unusual in these latter days to hear the champions of sanitary science boast, and justly boast, of the perceptible prolongation of life which has been secured through the beneficial effects of improved sanitary arrangements. But there was, he said, evidence of perceptible deterioration or degradation of life in the lower order of people. He had before him an analysis of the results of 32,324 examinations of men made by army surgeons from 1860 to 1864 inclusive.

A careful examination of these tables led to the inference that the lower class, from whom the recruits for the army are chiefly taken, are of an inferior physique now to what they were twenty-five years ago. The recruits drawn from town-bred populations gave by far the larger proportion of rejections, whilst the causes of rejections usually indicate a decidedly inferior physique.

The rejections from defective vision and diseases of the eyes were nearly 42 per 1,000, exclusive of all those whose defects of vision were so obvious as to attract the notice of the recruiter and who would be thus excluded.

There was a peculiar form of ophthalmia which, wherever it was met with, whether in military or civil life, was mainly caused by the vitiated atmosphere arising from over crowding. Of late years that scourge had been practically banished by the sanitary improvements that have been introduced into barracks. It was to his mind the most striking illustration of what such measures could accomplish, and there was no longer any excuse for the existence of the disease. As regards the question of epidemics properly so-called, they could hardly hope to meet the requirements of the case by any system of isolation. Quarantine in such cases was not only a waste of time, money and energy, it was also a cause of much unnecessary suffering to the sick and danger to the healthy.

The event of great interest in the section on

MEDICINE

was the address of the president of the section, Professor Gairdner.

The address dealt briefly with the various methods of healing, and then discussed at length the point whether the practice of medicine—that is, the art as distinguished from the science—had made a single step since Hippocrates. The progress of modern medicine since Cullen's time, he said, had been in the direction of stability not possessed by the art of medicine in any previous stage of its progress. As regards that great revolution in medical treatment of fevers and inflammations, which in some minds occupied in relation to medicine as advanced a position as antisepticism did in relation to surgery, he declared that while he had no doubt at all as to the advantage in some cases, and in a limited sense, of the so-called anti-pyretic or antithermic treatment, he had yet to be convinced of the advantage of its application as a routine in typhoid or in any other fever; but it might be readily conceded that both in typhoid fever and pneumonia, as previously treated in German hospitals, with an enormously high mortality, the method in question marked a distinct advance. But he had never ceased to hold, as a teacher of medicine, that all the fermentation of opinion, so to speak, with respect to current remedies and modes of treatment, all tokens of unrest and dissatisfaction, were in reality an advantage to the medical art, and tended both toward progress and stability. The prevention and cure of disease were not, indeed, identical; but the laws which guided the former were in a large measure and necessarily applicable to the latter. The same noxious influences which begot typhoid, for example, tended in a remarkable degree to exaggerate, if not to cause, a host of minor diseases. The same over crowding which was in a great degree the cause of typhoid fever, was a source in hospital wards of pyæmia and erysipelas, in barracks and jails of consumption (and this often among picked men), in certain endemic localities of contagious pneumonia, and probably of diphtheria.

It was impossible in the face of modern sanitation to escape the conclusion that the causes and remedies of disease alike were often to be sought more in the study of groups, and even of multitudes of cases, than the ordinary text-books or in other conventional remedies. In short, the treatment of diseases of every kind had been largely, even if silently, influenced by the thought that the chief disease-breeding factors must also be in individual cases adverse influences to be carefully studied and kept in check by those who would cure disease, and that in chronic cases especially, a large part of the cure consisted in the removal or modification of the injurious action of the causes.

In the section on

SURGERY,

Sir George Porter, presided.

The discussion on the radical cure of hernia, freely participated in, was chiefly confined to descriptions of various methods employed by the several gentlemen speaking.

Sir Spencer Wells read a paper in which he expressed a preference for Porro's operation over Cæsarian section. Dr. W. H. Allingham read a paper on "The Advantages of Inguinal Colotomy over the Lumbar Operation." The other work in the surgical section was not of special interest.

A communication from Dr. Playfair was read in the section on

OBSTETRICS, in which he maintained that all puerperal diseases are of septic origin. In the discussion Drs. Lawson Tait, Kidd and Mone-Madden maintained that some such cases are not septic.

Dr. Apostoli read a paper on "Electric Treatment of Fibroid Tumors," which was favorably discussed by Dr. Playfair and Sir Spencer Wells.

The paper by Dr. Sinclair, of Boston, U. S. A., on "Manual Dilatation" was very favorably received.

A number of cases were reported in the section on

PSYCHOLOGICAL MEDICINE, tending to show that treatment by galvanism may be curative in some forms of insanity.

The work in the other sections was not of special interest to American readers.

Professor Gairdner was elected president of the society for the next meeting, which will be held in Glasgow in 1888.

WM. B. MEANY, M. D.

LONDON, England.

